# Supplementary material for: Generation and Superposition of Perfect Vortex Beams in Terahertz Region via Single-Layer All-Dielectric Metasurface
Source: Nanomaterials (Basel). 2022 Aug 30;12(17):3010. doi: 10.3390/nano12173010 (PMC9457957; doi:10.3390/nano12173010)
Supplement: Supplementary file 1 [file nanomaterials-12-03010-s001.zip › nanomaterials-1879753-supplementary-update.pdf]

Supplementary Materials

Generation and Superposition of Perfect Vortex Beams in Terahertz Region via Single-Layer All-Dielectric Metasurface

Qi Wu [1,2], Wenhui Fan [1,2,3,*] and Chong Qin [1,2]

1 State Key Laboratory of Transient Optics and Photonics, Xi'an Institute of Optics and Precision Mechanics, Chinese Academy of Sciences, Xi'an 710119, China
2 School of Opto-Electronic Technology, University of Chinese Academy of Sciences, Beijing 100049, China
3 Collaborative Innovation Center of Extreme Optics, Shanxi University, Taiyuan 030006, China
* Correspondence: fanwh@opt.ac.cn

I. Polarization-independent properties

To verify the polarization-independent properties, the PVB generation of the device (d=4 mm, l=2) was simulated under the illuminations of five different polarizations, including horizontal and vertical, 45 degree, right- and left- circular polarization. The simulated results shown in Figure S1a and S1b reveal the similar ring radius for different polarizations.

II. The relationship between the ring radius and axicon period

The vortex beams are insensitive to the topological charge, but their sizes are related to the axicon periods (d) of the metasurface. In order to study the relationship between the ring radius and axicon period, twelve metasurface samples with identical topological charge l = 2 and different axicon periods from 0.5 mm to 7 mm are designed. The corresponding horizontal (black points) and vertical (red points) radii are plotted in Figure S1c, indicating the ring radius inversely proportional to the axicon periods

d. By fitting the curve, the specific numerical relationship can be obtained as:

$$r(d) = 11.30 * \exp\left(\frac{-d}{1.36}\right) + 1.51 \tag{S1}$$

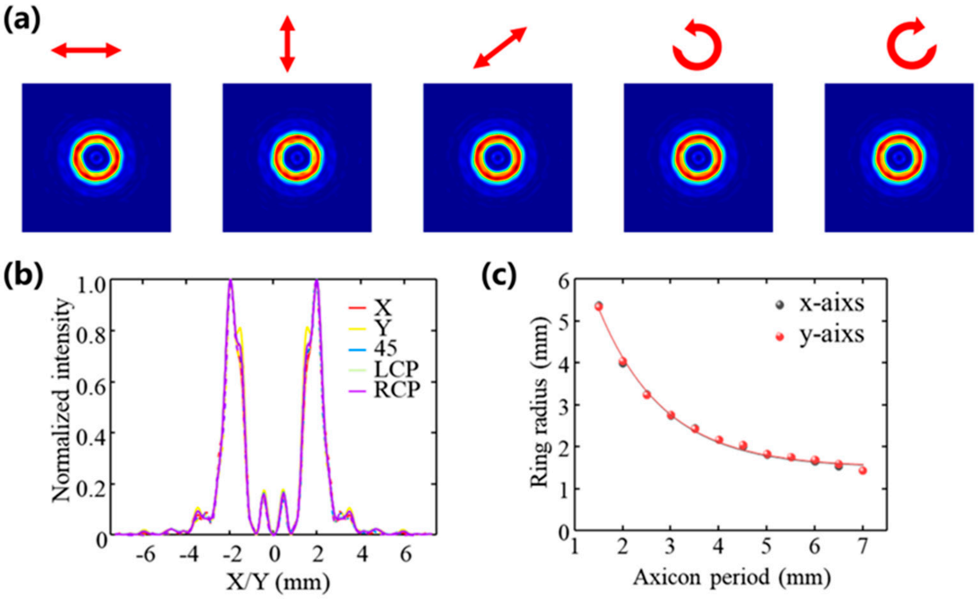

Figure S1. (a) The intensity of the PVBs at the focus point under different polarized incident light (depicted by the red double arrow). (b) Corresponding normalized cross-section of the annular intensity distribution along x-direction (solid line) and y-direction (dashed line). (c) The relationship between the ring radius and the axicon period with the unified parameters $l = 2$, $f = 14$ mm.

III. Bandwidth of the polarization-independent PVBs generator

We selected the meta-device with parameters of $d = 4$ mm and $l = 2$ to characterize the working bandwidth. As shown in Figure S2, we plotted the normalized annular intensity profiles of the PVBs with a frequency ranging from 0.65 THz to 0.9 THz at the focus plane, as well as plotting the

corresponding normalized cross-section of the annular intensity profiles along the x-direction (blue line) and y-direction (red line). The generation efficiencies have been calculated and marked in Figure S3 (b-g). According to these simulated results, we define the working frequency as the frequency when the generation efficiencies are larger than 40% and the intensity profile maintains a circular shape. Thus, the working bandwidth of our proposed meta-device is between 0.7 THz and 0.8 THz.

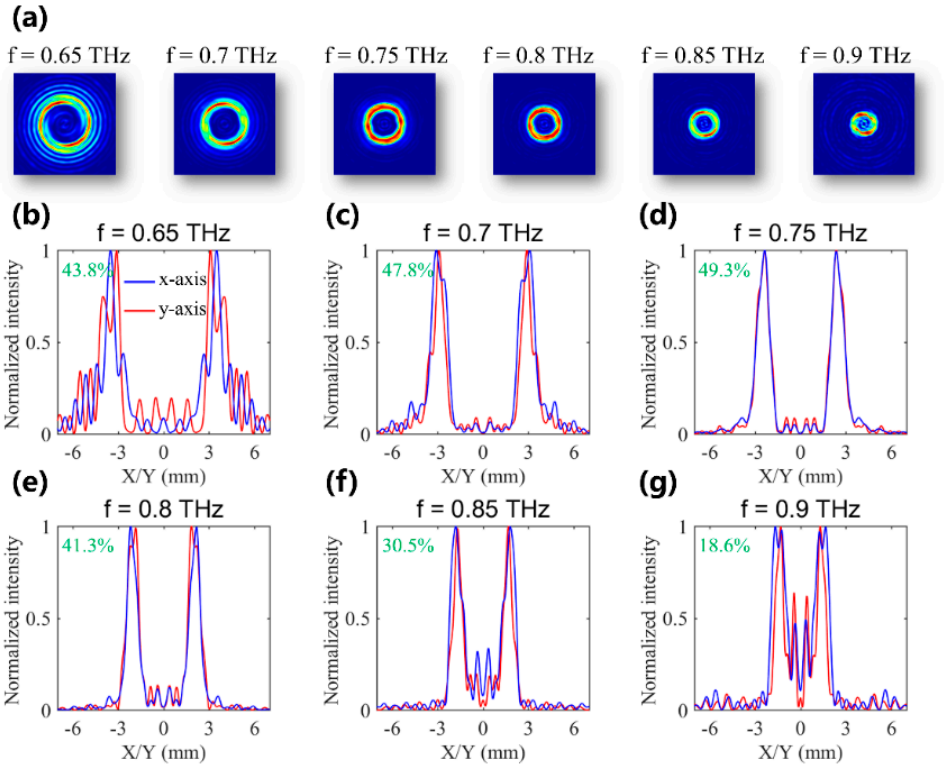

Figure S2. Characterization of the bandwidth of the polarization-independent PVBs generator. (a) The intensity of the PVBs at the focus point with a frequency ranging from 0.65 THz to 0.9 THz. (b-g) Corresponding normalized cross-section of the annular intensity distribution along x-direction (blue line) and y-direction (red line).

IV. Bandwidth of the spin-decoupled PVBs generator

As for the working bandwidth, we selected the spin-decoupled meta-device with parameters of $l_1 = l_2 = 3$, $d_1 = 4$ mm, $d_2 = 6$ mm to characterize. As shown in Figure S3a, we plotted the normalized annular intensity profiles of the PVBs with a frequency ranging from 0.6 THz to 1.0 THz at the focus plane for the LCP incidence. Figure S3 (b-g) plot the corresponding normalized cross-section of the annular intensity profiles along the x-direction (blue line) and y-direction (red line). The generation efficiencies have been calculated and marked in Figure S3 (b-g). According to these simulated results and working frequency definition, the working bandwidth of spin-decoupled meta-device is from 0.6 THz to 0.8 THz. In summary, the designed metasurface has good spin multiplexing performance, enhancing the device quality and its practical significance.

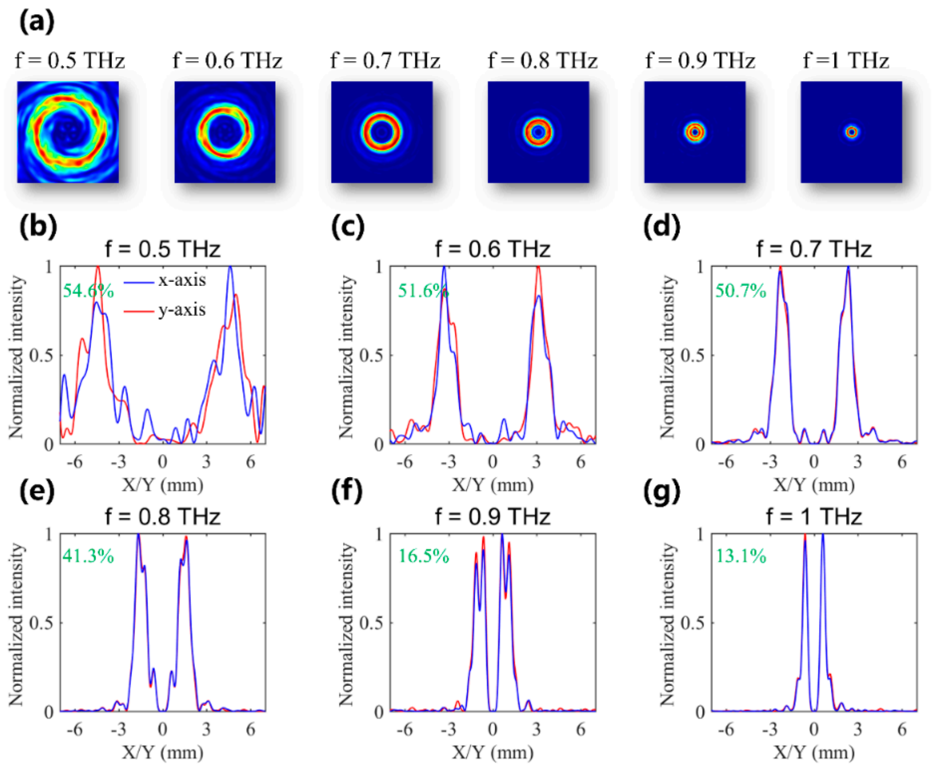

Figure S3. Analysis of the bandwidth of the spin-decoupled PVBs generator. (a) The intensity of the PVBs at the focus point with a frequency ranging from 0.5 THz to 1.0 THz. (b-g) Corresponding normalized cross-section of the annular intensity distribution along x-direction (blue line) and y-direction (red line).
